# Inhibiting Ferroptosis: A Novel Approach for Ulcerative Colitis Therapeutics

**DOI:** 10.1155/2022/9678625

**Published:** 2022-03-26

**Authors:** Jinke Huang, Jiaqi Zhang, Jinxin Ma, Jing Ma, Jiali Liu, Fengyun Wang, Xudong Tang

**Affiliations:** ^1^Department of Gastroenterology, Xiyuan Hospital of China Academy of Chinese Medical Sciences, Beijing, China; ^2^Department of Gastroenterology, Peking University Traditional Chinese Medicine Clinical Medical School (Xiyuan), Beijing, China; ^3^China Academy of Chinese Medical Sciences, Beijing, China

## Abstract

Ulcerative colitis (UC) is a recurrent and persistent nonspecific inflammatory bowel disease (IBD) that greatly affects human survival and social wealth. Despite the advances in the treatment of UC, there is still a high demand for novel therapeutic strategies for UC patients. Cell death is critical to the development and progression of UC. Understanding how intestinal cells die and how to prevent damage to intestinal cells is of great interest for the diagnosis and early treatment of UC. Ferroptosis, a novel form of regulated cell death (RCD) manifested by iron accumulation, lipid peroxidation, and excessive reactive oxygen species (ROS) production, has been shown to contribute to the development and progression of UC. Inhibitors of ferroptosis have been validated in models of UC. Here, we reviewed the mechanisms of initiation and control of ferroptosis and summarize the therapeutic activity of ferroptosis inhibitors in models of UC. We further discussed the possibility of inhibiting ferroptosis as a novel therapeutic target for UC. These findings revealed novel mechanisms to protect the colonic mucosa and highlighted the importance of ferroptosis in the disease process.

## 1. Introduction

Ulcerative colitis (UC) is a chronic nonspecific inflammatory bowel disease (IBD) characterized by abdominal pain, diarrhea, blood in the stool, and weight loss [1]. Worldwide, the prevalence of UC continues to rise, and its chronic recurrence and unpredictable nature lead to costly medical treatment, which imposes a huge economic burden on society [2]. The pathophysiological mechanisms of UC are still not well understood, and therefore, the efficacy of existing therapies is limited [3]. Further research on the pathogenesis of UC and the development of novel and effective therapeutic approaches are still urgently needed. Recently, regulated forms of cell death have been observed to be involved in the development and progression of IBD and have the potential to be novel therapeutic targets [4]. The first regulatory cell death modality identified was caspase-dependent apoptosis [5], which has accounted for the vast majority of cell death studies in recent decades. Autophagy, a process in which cells use lysosomes for self-digestion, is observed in both physiological and pathological processes of the organism, but whether it plays a positive or negative role has not been fully elucidated [6]. Ferroptosis is a novel form of programmed cell death mode distinguished from apoptosis, necrosis, and autophagy at the cellular morphology, biochemical characteristics, and genetic level, which is characterized by iron-dependent accumulation of lipid peroxidation to a lethal level [7–9].

As a unique and novel form of cell death, ferroptosis was initially observed in tumor cells [8]. As research has progressed, it has been found that the development and progression of a variety of diseases in addition to tumors are associated with ferroptosis [10–15]. Recent studies have identified ferroptosis as a key regulatory mechanism in intestinal diseases, and inhibition of ferroptosis is expected to be a new direction in the prevention and treatment of intestinal diseases [16–18]. Extensive ferroptosis has been reported to be observed in UC patients, and recent evidence has found that blocking ferroptosis significantly relieves UC symptoms and promotes intestinal repair [16, 19]. These findings imply a new understanding of the development of therapeutic strategies for UC. Therefore, to synthesize the role of ferroptosis in UC pathology and attempt to elaborate the possibility of targeting ferroptosis in UC therapeutic strategies, we conducted the present review.

## 2. Ferroptosis and Its Mechanism

**Figure 1:**
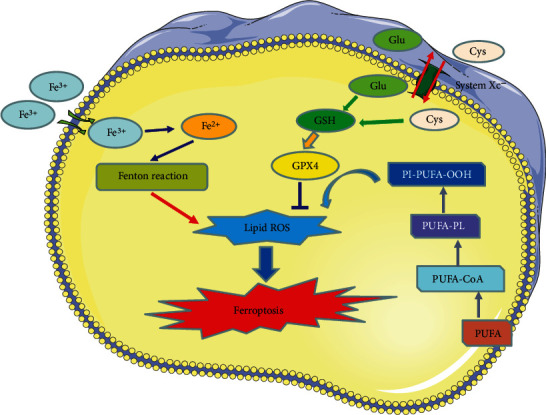
Ferroptosis is characterized by iron accumulation, lipid peroxidation, and excessive ROS production. The ferroptosis process is shown by this figure, including metabolic pathways, amino acid, lipid, and iron pathways which are showed in this figure.

Ferroptosis was formally defined in 2012 as nonapoptotic, iron-dependent cell death characterized by lipid peroxidation product accumulation and membrane polyunsaturated fatty acid (PUFA) depletion [9]. As an iron-catalyzed lipid peroxidation process, a large accumulation of reactive oxygen species (ROS) is the most prominent feature of ferroptosis, with sources including excessive production and insufficient scavenging [20, 21]. Details of the process of ferroptosis are shown in Figure 1, and markers that can be used for ferroptosis validation are outline in Table 1.

### 2.1. Iron Metabolism Pathway

Iron is one of the intrinsic elements of the body, and proper iron levels are essential to maintain normal physiological functions of the body, while once the free iron level exceeds the normal range, it can damage cells. Under physiological conditions, extracellular iron is in dynamic equilibrium with intracellular iron [22, 23]. Extracellular iron is imported into the cell as ferric ions (Fe^3+^) and is converted to ferrous ions (Fe^2+^) by intracellular endosomes before being transported to the cell membrane [13, 20]. Once the equilibrium is disrupted, intracellular Fe^2+^ is then in excess, and the excess Fe^2+^ and hydroxyl radicals can be directly catalyzed by Fenton reaction to produce large amounts of ROS, which promotes lipid peroxidation and induces ferroptosis [24, 25].

Intracellular iron overload is central to the stimulation of oxidative damage and iron sagging. Specifically, causes of intracellular iron overload are attributed to increased iron uptake by TfR1, decreased iron excretion by ferroportin, and increased free iron due to ferritin degradation [26]. Iron overload can also induce nonclassical iron uptake pathways causing ferroptosis [27, 28]. For example, CDGSH iron-sulfur structural domain 1 in mitochondrial membranes may contribute to the reduction of iron content and ROS production in mitochondria, thereby inhibiting the development of iron prolapse. However, the role of mitochondria in ferroptosis remains to be clarified [29].

Catalytic iron, namely, ferrous iron, mediates lipid ROS production via Fenton reaction and promote lipid peroxidation directly. Markers like iron content and ROS can be used for ferroptosis validation in this process.

### 2.2. Lipid Metabolism Pathway

The lipid bilayer structure of the cell membrane directly determines biofilm properties and is essential for maintaining the integrity of membrane function. Increasing evidence suggest that lipid peroxidation is the driving force of ferroptosis [30, 31]. As the key component of cell membranes, polyunsaturated fatty acids (PUFAs), especially arachidonic acid (AA) and epinephrine (AdA), are preferentially oxidized by reactive free radicals [31]. Phospholipids containing PUFAs are lipid precursors for peroxidation reactions, and exposure to exogenous PUFAs can increase ferroptotic sensitivity [31]. Conversely, supplementation with deuterated PUFAs or exogenous MUFAs decelerated the accumulation of lipid peroxides and thus protected cells from ferroptosis [21, 31].

Phosphatidylethanolamine (PE) containing AA or AdA has been reported to be oxidized to phospholipid hydroperoxides (PE-AA/AdA-OOH) via a nonenzymatic reaction, which triggers ferroptosis [31]. In the presence of intracellular ferrous overload, ROS may be converted to hydroxyl radicals (HO˙), which subsequently affect PUFA on the cell membrane [32]. Free PUFAs are oxidized via the catalytic pathways of lysophosphatidylcholine acyltransferase 3 (LPCAT3), acyl-CoA synthetase long-chain family member 4 (ACSL4), and lipoxygenases (LOXs) [21, 33]. ACSL4 and LPCAT3 have been demonstrated to be key regulators of PUFA-PL biosynthesis [31]. PUFA can be acetylated by ACSL4 to form PUFA-CoA, followed by LPCAT3 insertion of PUFA-CoA into lysophospholipids to form PUFA-PL [34]. Enzymatic lipid peroxidation of ferroptosis is regulated by the LOXs and is predominantly dominated by LOX5 and LOX12/15 [35].

Notably, GPX enzymes inhibit the oxidation of lipids, particularly GPX4, which limits the formation of reactive lipid alkoxides and reduces phospholipid hydroperoxides to lipid alcohols [11].

Lipid peroxide-induced ferroptosis can be summarized in the following three procedures (Figure 1). First, ACSL4 catalyzes the esterification of AA or AdA to PE. Second, LPCAT3 generates PUFA-PE based on PE substrates. Finally, 15-LOX oxidizes AA-PE and AdA-PE to ferroptotic signals (PE-AA-OH and PE-AdA-OOH) [36].

### 2.3. Amino Acid Metabolism Pathway

As a member of the glutathione peroxidase family, GPX4 catalyzes the conversion of free hydrogen peroxide to water or the reduction of cytotoxic lipid hydroperoxides (L-OOH) to nontoxic lipid alcohols (L-OH), thereby inhibiting the production of lipid ROS [37, 38]. Thiol-containing tripeptide glutathione (GSH) is the major antioxidant in mammalian cells and the primary substrate of GPX4. The conditions leading to glutathione depletion directly affect the activity and stability of GPX4, thus rendering the cells more susceptible to ferroptosis [39]. GSH prevents lipid peroxidation of polyunsaturated fatty acids in cell membranes via GSH-Px, whereas inhibition of the uptake of GSH-generating substrates causes lipid peroxidation to occur [39].

Both the metabolism and synthesis of amino acids are associated with the process of ferroptosis [40]. Glutamine can be converted to glutamate by glutaminases (GLS1 and GLS2) [41]. High extracellular glutamate concentrations promote intracellular cystine depletion and eventually lead to GSH depletion and GPX4 inactivation, with ferroptosis triggered as a result [42] *Α*-ketopentanoic acid is a product of glutamine-driven intracellular metabolic pathways and can also have a glutamine-like effect when ferroptosis occurs [43]. In addition, the glutaminase GLS2, a transcriptional target of the tumor suppressor p53, can inhibit iron sag by limiting DPP4-mediated lipid peroxidation [44, 45].

## 3. Ferroptosis in Ulcerative Colitis

**Figure 2:**
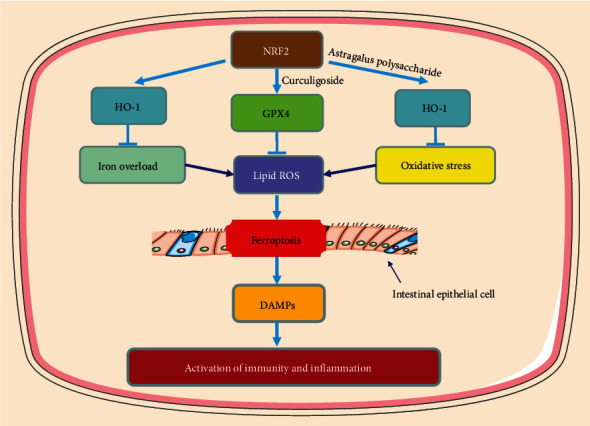
Role of ferroptosis in UC. Ferroptosis promotes the release of damage-associated molecular patterns (DAMPs) from the intestinal epithelium. Subsequently, the immune response is hyperactivated, leading to intestinal inflammation and epithelial damage.

The basic features of iron sagging include iron deposition, lipid peroxidation accumulation, GSH depletion, GPX4 inactivation, and LOX upregulation, all of which have been elucidated to be associated with the pathogenesis of UC [16, 46–49]. Iron chelators have been reported to significantly reduce ROS accumulation, improve clinical symptoms, and promote intestinal epithelial cell repair in UC patients [50, 51], and conversely, high dietary iron supplementation can exacerbate UC symptoms [52, 53]. Sensitivity to erastin-induced ferroptosis was found in a UC cell model and could be rescued by a ferroptosis specific inhibitor [16, 46–49]. The ferroptosis phenomenon has also been observed in mouse models of UC and can reduce symptoms by targeted inhibition of ferroptosis [16, 46–49]. These findings further validate that inhibition of ferroptosis may be a novel target for alleviating UC. Details of the role of ferroptosis in UC are shown in Figure 2.

### 3.1. The Role of Iron in UC

In UC, excessive ROS production by the colorectal mucosa may cause changes in cellular proteins, lipids, and nucleic acids, leading to several cellular dysfunctions that may affect the disease process [54]. Excess-free iron can aggravate oxidative activity within the intestinal epithelium through multiple mechanisms. First, hydrogen peroxide and ferrous ions can generate large amounts of ROS directly through the Fenton reaction. Moreover, superoxide can promote the conversion or release of ferrous ions, which in turn promotes the Fenton reaction [51]. Recessive mutations in the hemochromatosis gene (Hfe) are strongly associated with iron overload [55]. Hfe knockout mice were observed to have increased MDA in colonic tissue [56] and exhibited more severe symptoms of colonic mucosal injury, such as hematochezia and diarrhea [57]. In addition, iron overload not only leads to dysregulated ROS generation and interferes with intestinal bacteria, which in turn aggravates enteritis [58]. While the phenomenon of ferroptosis has been found in UC, accompanied by iron overload, the use of iron chelators can significantly reduce ROS and improve colitis symptoms [16, 46–51]. Therefore, these studies all highlight the pathological role of iron overload in the development of UC; that is, the deposition of iron in the intestine leads to severe oxidative stress (OS), promotes the production of ROC through the Fenton reaction, triggers ferroptosis, and then stimulates the release of damage-associated molecular patterns (DAMPs) to cause intestinal immune and inflammatory responses. Iron chelation, on the other hand, may be a promising therapeutic strategy for UC.

### 3.2. The Role of Oxidative Stress in UC

OS is considered to be a potential driver of UC induction and progression and has been well reported in both patients and animal models of UC [60–63]. A higher OS status is thought to be a cause of altered immune and inflammatory responses that contribute to the development of UC [64, 65]. With the development of UC, the activity of inflammatory cells in the colon is greatly increased, leading to increased production of pro-oxidant molecules [60]. OS is attributed to redox imbalance, which is caused by excessive production of ROS and inadequate response of the antioxidant system to eliminate ROS [66]. Cytokine-induced elevated levels of myeloperoxidase also lead to ROS production [67]. Excessive ROS production leads to changes in cellular proteins, lipids, and nucleic acids, resulting in several cellular dysfunctions that may affect the course of UC. Colonic epithelial cells contain several antioxidant systems, such as antioxidant enzymes, namely, GSH, GPX4, and LOXs [68], but they are usually dysregulated in UC pathological conditions. As mentioned above, dysregulation of OS function is also observed in iron hypoplasia; it is hard to believe that the broad similarities between UC pathology and aspects of the ferroptosis cell death pathway are merely coincidental.

GSH can directly scavenge ROS and enhance cellular antioxidant capacity [69]. However, higher OS can promote GSH depletion and reduce GSH synthesis [70]. The depletion of GSH is widely observed in UC patients and experimental animal models of UC [71, 72]. Inhibition of the synthesis of GSH has been found to result in intestinal epithelial cell injury, while GSH supplementation significantly improves colonic health [73]. GPX4 is an important antioxidant enzyme that plays an important regulatory role in ferroptosis [74]. Inactivation of GPX4 promotes lipid peroxidation and induces ferroptosis [42]. Studies have reported that Nrf2-Gpx4 signaling pathway can significantly inhibit ferroptosis [75, 76], but Nrf2-Gpx4 signaling pathway is inhibited in UC [16, 46, 77]. Activation of Gpx4 can markedly inhibit ferroptosis and improve UC symptoms [46, 47]. LOXs are able to promote lipid hydroperoxide production and drive ironophilic cell death [31]. Alox15 deletion has been reported to promote inflammation suppression, intestinal barrier integrity maintenance, and colonic injury, while Alox15 overexpression exhibits more severe symptoms of colitis [78]. As a master regulatory molecule of 15-LOX, it was observed that the loss of phosphatidylethanolamine-binding protein 1 (PEBP1) is beneficial to reduce colitis symptoms and accelerate mucosal recovery [79]. Similar effects have been observed with other selective inhibitors acting on 5-LOX [80, 81].

### 3.3. The Role of Other Ferroptosis Regulators in UC

Nrf2, a major regulator of the antioxidant response, induces the expression of endogenous antioxidant proteins responsible for blocking lipid peroxidation to alleviate OS. Recent studies have demonstrated that Nrf2 can protect UC by inhibiting ferroptosis [19, 46, 49, 77]. Nrf2 has been reported to be involved in the regulation of ferroptosis by controlling the expression of quinone oxidoreductase 1, iron metabolism proteins, GPX4, and GSH production [82, 83]. Nrf2 has been widely demonstrated to be involved in the pathophysiological processes of UC. In NRF2 knockout UC mice, a significant increase in the severity of colitis and risk of colitis-associated colorectal cancer was observed [84, 85]. The regulatory effect of NRF2 on ferroptosis was also observed, with activation of NRF2 inhibiting ferroptosis and knockdown of NRF2 increasing sensitivity to various ferroptosis inducers [86, 87]. Notably, compounds that activate NRF2 inhibit ferroptosis and attenuate colitis-related mucosal damage and colonic inflammation [88]. However, the response of Nrf2 to OS is not specific to ferroptosis, as Nrf2 is also associated with the regulation of pyroptosis [89]. This suggests that the protective effect of Nrf2 on UC may be related to the regulation of multiple forms of cell death.

Mutations in the tumor suppressor P53 have been associated with the development of UC and UC-associated colorectal cancer [90]. Furthermore, P53 transcription inhibits the expression of the cystine/glutamate reverse transporter protein subunit SLC7A11, which in turn disrupts GSH production, thereby sensitizing cells to ferroptosis [73]. It has been observed that P53 is also involved in the regulation of ferroptosis by a mechanism that lies downstream of activation of arginine/arginine N1-acetyltransferase 1 and Alox15 or through transcriptional upregulation of mitochondrial glutaminase 2 [43, 91].

## 4. Conclusion and Future Perspectives

Patients with UC have a high demand for novel therapeutic strategies. Ferroptosis is a form of RCD characterized by iron overload, lipid peroxidation GPX4 inactivation, and GSH depletion. Based on these features, it is difficult to believe that the broad similarities in UC pathological features and ferroptosis pathways are merely coincidental. Ferroptosis plays an important role in the pathogenesis and progression of UC. The main features of ferroptosis have been widely observed in the colonic tissue of UC patients and animal models. By using UC mouse models, genetic or pharmacological manipulation of ferroptosis-associated genes can improve symptoms and promote recovery from experimental colitis. More specifically, a number of potent ferroptosis regulators (Table 2) can resist lipid oxidation and promote repair of intestinal damage in UC. Targeted inhibition of iron sagging may be a potential novel therapeutic strategy for UC.

Notably, targets involved in the regulation of ferroptosis are continuously being explored. Frataxin protein, which localizes to mitochondria and is involved in the biosynthesis of iron-sulfur clusters, has recently received the attention of investigators. Decreased frataxin expression can lead to iron accumulation at the mitochondrial level, uncontrolled production of reactive oxygen species, and lipid peroxidation [93]. These features are also common to ferroptosis. It has been reported that suppression of frataxin expression specifically activated iron starvation stress, accelerated free iron accumulation, enhanced lipid peroxidation, and resulted in ferroptosis [94–96]. Conversely, enforced expression of frataxin blocked the iron starvation response and erastin-induced ferroptosis [94, 95]. Hence, frataxin is considered to be a key regulator of ferroptosis by modulating iron homeostasis and mitochondrial function. There is a lack of evidence for the role of frataxin in UC. Since frataxin has a key regulatory role on ferroptosis, it may affect UC by regulating ferroptosis, but this hypothesis needs to be further validated by future studies.

Ferroptosis mainly occurs in IE mediating the pathogenesis and development of UC. Based on the currently available evidence, we believe that ferroptosis is a negative regulator of UC, and inhibition of ferroptosis is expected to be a novel approach for UC therapeutics. Although various inhibitors for ferroptosis have been observed to have positive effects in attenuating tissue damage associated with colitis, the underlying molecular mechanisms remain elusive, and further molecular mechanism studies are important for identifying more selective ferroptosis modulators. Furthermore, it is uncertain whether intestinal immune cells undergo ferroptosis in addition to IECs, and exploring ferroptosis in specific types of epithelial cells and intestinal immune cells will help to comprehensively understand the effects of ferroptosis on UC and provide new evidence for clinical decision-making in UC.

## Figures and Tables

**Table 1:** Markers that can be used for ferroptosis validation.

Metabolic processes	Markers
Iron metabolism pathway	Fe^3+^, Fe^2+^
Oxidative stress	ROS, SOD, OH, H_2_O_2_
Lipid metabolism pathway	MDA, LPO
Amino acid metabolism pathway	GPX4, GSH, GR, GLU, Cys

**Table 2:** Validated ferroptosis regulators in the UC model.

Gene/axis/compound	Mechanism	Function
Ferrostatin-1 [19, 92]	Downregulation of PTGS2 levels, MDA levels, and iron content	Inhibition
Furin [46]	Activation of Nrf2 and upregulation of Gpx4 expression	Inhibition
MELK [48]	Inhibited ferritin formation in intestinal tissues	Inhibition
Lip-1 [19]	Blocking Nrf2/HO-1 and reducing the levels of COX2, ACSL4, FTH1	Inhibition
Deferprone [19]	Blocking Nrf2/HO-1 and reducing the levels of COX2, ACSL4, FTH1	Inhibition
NF-*κ*Bp65 [16]	Inhibition of endoplasmic reticulum stress	Inhibition
Curculigoside [47]	Induction of GPX4	Inhibition
Fer-1 [16]	Reduces MDA, iron, and FTH levels	Inhibition
Astragalus polysaccharide [49]	Inhibiting NRF2/HO-1 pathway	Inhibition
RSL3 [16]	Accumulation of ROS	Induction

## Data Availability

All data obtained or analyzed during this work are included within the article.
